# Molecular Modeling in Anion Exchange Membrane Research: A Brief Review of Recent Applications

**DOI:** 10.3390/molecules27113574

**Published:** 2022-06-02

**Authors:** Mirat Karibayev, Sandugash Kalybekkyzy, Yanwei Wang, Almagul Mentbayeva

**Affiliations:** 1 Department of Chemical & Materials Engineering, School of Engineering and Digital Sciences, Nazarbayev University, Nur-Sultan 010000, Kazakhstan; mirat.karibayev@nu.edu.kz; 2 Laboratory of Advanced Materials and Systems for Energy Storage, Center for Energy and Advanced Materials Science, National Laboratory Astana, Nazarbayev University, Nur-Sultan 010000, Kazakhstan; sandugash.kalybekkyzy@nu.edu.kz; 3 Laboratory of Computational Materials Science for Energy Applications, Center for Energy and Advanced Materials Science, National Laboratory Astana, Nur-Sultan 010000, Kazakhstan

**Keywords:** anion exchange membrane, fuel cell, transportation mechanism, chemical stability, modeling, multi-scale

## Abstract

Anion Exchange Membrane (AEM) fuel cells have attracted growing interest, due to their encouraging advantages, including high power density and relatively low cost. AEM is a polymer matrix, which conducts hydroxide (${\mathrm{OH}}^{-}$) ions, prevents physical contact of electrodes, and has positively charged head groups (mainly quaternary ammonium (QA) groups), covalently bound to the polymer backbone. The chemical instability of the quaternary ammonium (QA)-based head groups, at alkaline pH and elevated temperature, is a significant threshold in AEMFC technology. This review work aims to introduce recent studies on the chemical stability of various QA-based head groups and transportation of ${\mathrm{OH}}^{-}$ ions in AEMFC, via modeling and simulation techniques, at different scales. It starts by introducing the fundamental theories behind AEM-based fuel-cell technology. In the main body of this review, we present selected computational studies that deal with the effects of various parameters on AEMs, via a variety of multi-length and multi-time-scale modeling and simulation methods. Such methods include electronic structure calculations via the quantum Density Functional Theory (DFT), ab initio, classical all-atom Molecular Dynamics (MD) simulations, and coarse-grained MD simulations. The explored processing and structural parameters include temperature, hydration levels, several QA-based head groups, various types of QA-based head groups and backbones, etc. Nowadays, many methods and software packages for molecular and materials modeling are available. Applications of such methods may help to understand the transportation mechanisms of ${\mathrm{OH}}^{-}$ ions, the chemical stability of functional head groups, and many other relevant properties, leading to a performance-based molecular and structure design as well as, ultimately, improved AEM-based fuel cell performances. This contribution aims to introduce those molecular modeling methods and their recent applications to the AEM-based fuel cells research community.

## 1. Introduction

The recent data obtained from the available literature confirmed that the global energy consumption for renewable energy sources, including solar, wind, geothermal, and bio-energies, increased from 0.1% to 4.1% [1]. In contrast, petroleum, gas, and coal energy sources fluctuated by around 30%. between 1978 and 2018 [1]. There were two known and main pathways for using solar energy, such as (i) storing in the form of a chemical bond as hydrogen fuel and (ii) directly converting into electricity. Remarkably, there was a wide range of available sources for hydrogen fuel production, including water, natural gas, glycerol, biomass, and others [2]. Hydrogen is a non-carbon-based energy source and clean-energy carrier, with a higher energy density than petroleum [2]. However, there are three main challenges, around hydrogen fuels as a renewable energy source and related large-scale applications [3].

A fuel cell is an electrochemical cell or device that enables the conversion of chemical energy to electrical energy [4]. There are various types of fuel cells, including alkaline fuel cells (AFCs)—mostly related to anionic exchange membranes (AEMs) [5,6,7,8], proton-exchange membrane fuel cells (PEMFC), also known as polymer electrolyte membrane (PEM) fuel cells [5,9,10,11], phosphoric acid fuel cells (PAFCs) [12], molten carbonate fuel cells (MCFCs) [13], solid oxide fuel cells (SOFCs) [14,15], enzymatic (bio)fuel cells (EFCs) [16], direct methanol fuel cells (DMFCs) [17], and others.

**Figure 1:**
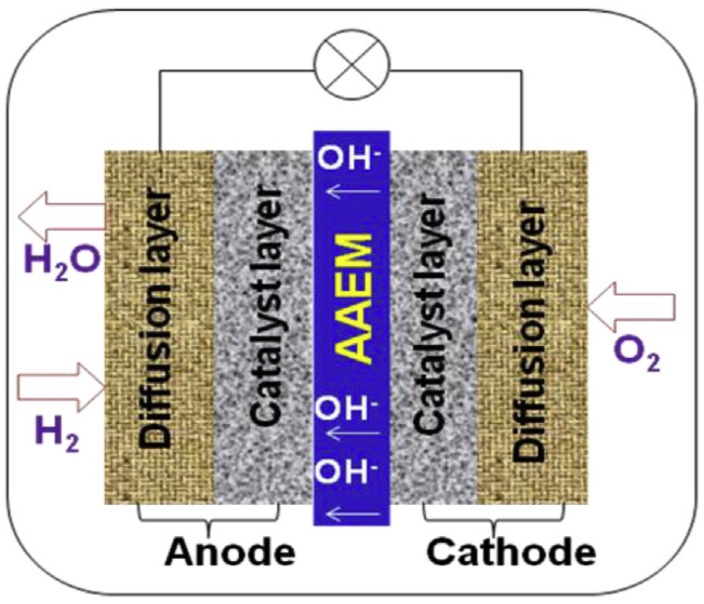
Schematic illustration of the working principle of AEMFCs. Reprinted with permission from [19]. Copyright 2015, for Elsevier.

This review focuses on anion exchange membrane (AEM) fuel cells (AEMFCs). AEMFCs are attracting ever-increasing attention and are promising electrochemical devices for energy production, due to their low production cost, high ${\mathrm{OH}}^{-}$ ion conductivity, low operation temperature, and high power density [7,8,18,19]. AEMFCs employ a solid (often polymeric), hydroxide conductive membrane as their electrolyte (Figure 1) [19]. The membrane electrode assembly (MEA) part of the AEMFC is sandwiched between the anode and cathode plates [20]. As can be seen in Figure 1, the MEA part is composed of gas diffusion layers (GDL), catalyst layers (CL), and AEM [20]. The GDL layers consist of the backing and the micro-porous layers [20]. The CLs are combinations of electro-catalysts and an ionomer, which results in the triple-phase boundaries formation for electrochemical reactions, such as a hydrogen oxidation reaction (HOR, Equation (1)) and an oxygen reduction reaction (ORR, Equation (2)) [20].

AEM is a polymer matrix, where cations, such as quaternary ammonium (QA) head groups, are bound to the polymer backbone and responsible for the conduction of hydroxide (${\mathrm{OH}}^{-}$) ions [21]. AEMs play a prominent role in addressing alkaline-fuel-cell efficiency and cost [18]. The main function of AEMs is to transport ${\mathrm{OH}}^{-}$ ions, obtained at the cathode, to the anode, where ${\mathrm{OH}}^{-}$ ions electrochemically react with hydrogen fuel to release electrons, as shown below:

(1) $$\mathrm{At}\;\mathrm{anode}:H_{2}+2{\mathrm{OH}}^{-}⟶2H_{2}O+2e^{-}$$

(2) $$\mathrm{At}\;\mathrm{cathode}:O_{2}+2H_{2}O+4e^{-}⟶4{\mathrm{OH}}^{-}$$

In AEMFCs, hydrogen as a fuel is delivered to the anode; oxygen and water are supplied to the cathode. Oxygen reduces at the cathode part, and the fuel oxidizes at the anode part. ${\mathrm{OH}}^{-}$ ions are transported via the electrolyte toward the anode from the cathode. Delivered ${\mathrm{OH}}^{-}$ ions react with the fuel, to produce water and electrons at the anode. Finally, electrons undergo the circuit to yield current [18,20].

### 1.1. Challenges in AEM-Based Fuel Cells

To the best of our understanding, there are four main challenges that need to be solved to achieve large-scale commercialization of AEMFCs [18,19,22]:

(i) The chemical degradation of the QA head groups of the AEM matrix creates a barrier to cell performance stability.
(ii) Enhancing ${\mathrm{OH}}^{-}$ ion diffusivity is required.
(iii) There is a necessity for effective and non-precious catalysts, for HOR and ORR, in an alkaline medium.
(iv) The implementation of ambient air feed leads to carbonation issues.

**Figure 2:**
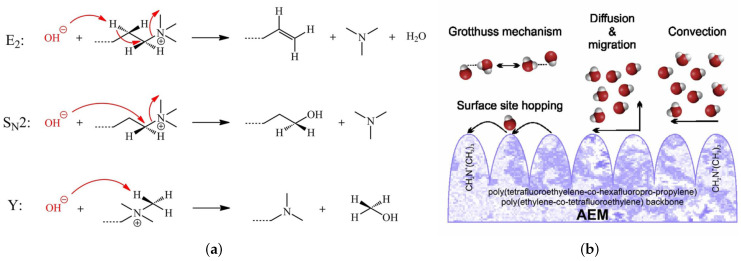
(**a**) Possible degradation mechanisms of the QA head groups in alkaline conditions, such as Hofmann elimination ($E_{2}$), nucleophilic substitution ($S_{N}2$), and ylide formation (Y); (**b**) the five transportation mechanisms of ${\mathrm{OH}}^{-}$ ion in AEM. Reprinted with permission from [34,35]. Copyright 2017, for John Wiley and Sons, [34] and copyright 2018, for Elsevier [35].

Concerning the low chemical stability under alkaline conditions, due to the degradation of the QA head groups, three different degradation mechanisms of the QA head groups of AEMs at high pH and low hydration level (HL) were proposed, including Hofmann elimination, nucleophilic substitution, and ylide formation, as shown in Figure 2a [19]. The HL is defined as the number of water molecules per the QA head group ($\lambda \equiv n_{H_{2}O}/n_{\mathrm{QA}}$). At the same time, the working principle of AEMFCs results in gradients in the cell that could lead to low-hydration conditions (λ≤4) within the cell. Moreover, the degradation of the QA head groups, usually, took place under low HL. Therefore, it is highly important to study the chemical stability of the QA head groups and ${\mathrm{OH}}^{-}$ ion transportation at the lower hydration level. Nonuniform water distribution exists at a low hydration level. At the same time, layered water structures were observed at a high hydration level (λ≥11) [19,23,24,25,26,27]. Figure 2b illustrates the possible transport mechanisms observed in AEMs, including Grotthuss behavior, diffusion, convection, surface site hopping, and vehicular mechanisms [25,26,28,29,30,31,32,33]. The degradation mechanisms of the QA head groups under high pH and the transportation mechanism of ${\mathrm{OH}}^{-}$ ions in AEMs are, still, active topics of research.

There are several types of ${\mathrm{OH}}^{-}$ ion transportation mechanisms in AEMFCs. The Grotthuss mechanism is the first type of transportation mechanism, which occurs in the bulk of water molecules at a high hydration level. As a result, the AEM’s ion exchange capacity (IEC) will reach its highest value [34]. Surface site hopping is the second type of transportation mechanism, which includes the diffusion of ${\mathrm{OH}}^{-}$ ions by successive hops from one side of the the QA head group to another, in the presence of strong electrostatic attractive forces and low HL. As a result, the IEC will be very low during the surface-site-hopping mechanism [25,26,28,29,30,31,32,33,35,36]. Convective transportation is the third type of transportation mechanism, which notes when ${\mathrm{OH}}^{-}$ ions capture water molecules, by the formation of hydrogen bonds and, thus, generate convection flow in the presence of a pressure gradient, between the boundaries of the anode and cathode diffusion layers of the fuel cell (FC) and the MEA [36,37]. Diffusion & migration transportation is the fourth transportation mechanism, which takes place in the bulk of water molecules, in the presence of a gradient in concentration or electric potential [37,38]. Finally, a vehicular mechanism is the fifth transportation mechanism, which occurs when the polymeric matrix of AEM moves like a vehicle to transport ${\mathrm{OH}}^{-}$ ion [32,38,39].

In addition, phase-segregated AEM structures can, also, affect the transportation of ${\mathrm{OH}}^{-}$ ions [32,33,40,41,42,43,44,45,46]. Two known and important phase-segregation structures exist in AEM, including the lamellar structure and the confined structure. The positively charged QA head groups of AEM could strongly phase, separated into two distinct morphologies, such as lamellae and confinement to allow for the transportation of ${\mathrm{OH}}^{-}$ ion. The lamellae and confined structures are critically important structures of emerging electrochemical devices. Therefore, understanding the water structure and behavior of ions in these lamellae and confined structures is essential to studying the chemical stability of the QA head groups and transportation of ${\mathrm{OH}}^{-}$ ion in AEM. In addition, the morphology of AEM is, mainly, controlled by the hydration level, degree of quaternization, and backbone composition [32,33,40,41,42,43,44,45,46].

The experimentally fabricated materials for AEM applications are, usually, characterized by their chemical, thermal, and mechanical stability performances, as well as their ${\mathrm{OH}}^{-}$ ion conductivity and ion exchange capacity [21,36]. The materials characterization techniques for testing the mechanical properties of AEM might be tensile testing, water uptake, swelling ratio, and thickness. UV-visible spectroscopy, X-ray powder diffraction, Fourier-transform infrared spectroscopy, scanning electron microscopy, and transmission electron microscopy determine the chemical structure of AEM. Thermogravimetric analysis completes the thermal characterization [21,36]. Nowadays, despite the progress of experimental investigations for chemical and mechanical stability, the measurement of the IEC of AEMFCs, using the advanced characterization methods mentioned above, is still a challenge, for studying the detailed degradation mechanisms and chemical stability of cationic head groups, ${\mathrm{OH}}^{-}$ ion transportation mechanisms, and the mechanical stability of AEMFCs.

### 1.2. Molecular Modeling and Simulations

**Figure 3:**
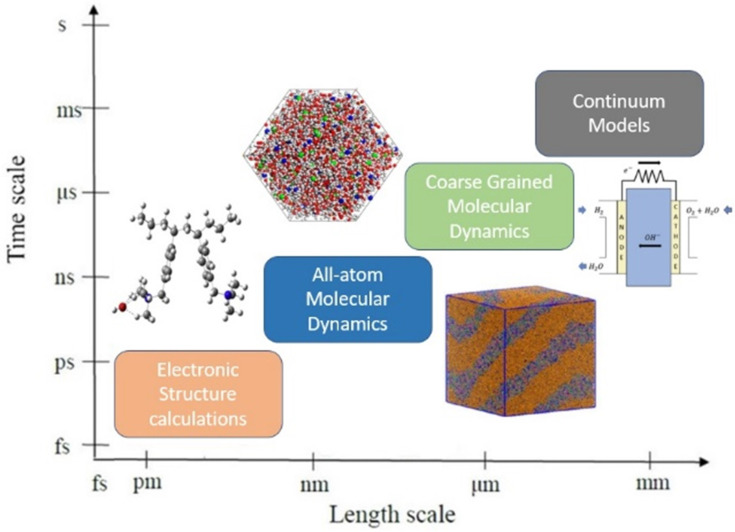
The level of hierarchy from the atomic scale to the system level. Reprinted with permission from [50,51,52,53]. Copyright 2016, for Elsevier [50], copyright 2017, for the American Chemical Society [51], copyright 2014, for the American Chemical Society [52], and copyright 2017, for Elsevier [53].

The development of physical theories as well as physics and/or data-based modeling and simulation techniques could help us guide and rationalize the design of AEMFCs. The main goal of physical theories is to explain the observations obtained from experimental studies [19]. At this stage, a mathematical model or computational model is a simplified representation of a designed system to mimic reality, with some assumptions and approximations that enable us to describe, investigate, and predict properties of interest and performance of the designed system, to solve the above-stated three challenges [19]. The rapid advances in computational resources have enabled researchers to apply a variety of computational molecular and materials modeling and simulation methods (see Figure 3), ranging from the electron and atom levels to membranes, electrodes, and even fuel cells. In favor of open and transferable research, computational studies have been stored in many repositories and databases. In recent years, material informatics has emerged as a new field, which helps us choose the desired property of our designed material from large material datasets [47,48,49].

There have already been a number of excellent reviews and perspective articles on the applications of molecular and materials modeling methods, in advanced energy materials research. We recommend the following articles [54,55,56,57,58,59,60,61,62,63,64,65,66,67] to interested readers. There is a wide range of applications of such molecular & materials modeling methods, at different scales, to study drug design, biological processes, wastewater treatment, enhanced oil recovery, concrete admixtures, rational design of carbon nano-sheets, lithium-ion batteries, and fuel cells, to name a few [68,69,70,71,72,73,74,75,76,77,78,79,80,81,82,83,84,85].

This review focuses on recent molecular modeling and simulation studies, devoted to AEM-based fuel-cell research, spanning a broad range of length and time scales, as schematically shown in Figure 3. At this stage, modeling and simulations at a different scale could provide deeper mechanistic information, to shed light on understanding the chemical and mechanical stability as well as the transportation of ${\mathrm{OH}}^{-}$ ions. Furthermore, the modeling and simulation have become faster, more accurate, and predictive of materials, mechanisms, and processes, at different timescales and lengths, as illustrated in Figure 3 [86,87]. Figure 3 illustrates the modeling and simulation in material design, which consists of methodologies ranging from quantum mechanics (QM) to atomistic/molecular-level simulations to the mesoscale level and to continuum-physics-based methods [88].

### 1.3. Outline of This Review

In this work, the topical and characteristic analysis were implemented to review the recent studies of AEMFC, via modeling and simulation, at the different scale. Computational studies at the different scale have been widely implemented among researchers. Modeling and simulations at the different scale has become a powerful tool to investigate the chemical stability of the QA head group-based polymeric matrix of AEM and transportation of ${\mathrm{OH}}^{-}$ ions in AEMFCs. There are many examples of typical AEMFCs models:

- The electronic structure (ab initio) calculation models developed for providing fundamental insights into the processes governing local properties, such as degradation mechanisms and the chemical stability of the QA head groups.
- Ab initio MD simulations developed to investigate the vehicular transportation mechanism of ${\mathrm{OH}}^{-}$ ions and the confined structure of AEM.
- All-atom molecular dynamic (MD) simulations developed to investigate the transportation mechanism of ${\mathrm{OH}}^{-}$ ion, and nanophase segregation of AEM matrix.
- Coarse-grained MD (CGMD) simulations developed to study mesoscale segregation, and the transport mechanism of ${\mathrm{OH}}^{-}$ ions of polymeric AEM matrix.
- A range of continuum models developed to study finite element analysis (FEA), fluid dynamics, and reaction-rate calculations, based on rate theories in AEMFCs.

To the best of our knowledge, few attempts have performed to review all aspects of modeling and simulations for AEMFCs systems [89]. This contribution aims to introduce those molecular modeling methods and their recent applications to the AEM-based fuel cells research community.

The contents discussed in this paper are illustrative, while the examples given are representative.

We believe that this review will be helpful to prompt researchers, working on the design of AEMFCs, to think about electronic structure calculations, classical all-atom MD simulations, and CGMD simulations, when studying the chemical stability and ${\mathrm{OH}}^{-}$-ion-transportation properties of AEMFCs.

## 2. Electronic Structure Calculations Based on the Density Functional Theory (DFT)

In ab initio calculations, the ground state for a set of atoms is obtained by solving the Schrodinger equation. The time-independent Schrodinger equation for the collection of many atoms is the basis of quantum mechanics (QM) [90,91,92,93,94,95,96,97,98,99,100,101,102,103,104,105,106,107]. Most of the computational studies on the chemical stability of AEM study via DFT calculations. The following paragraphs discuss various computational DFT studies about AEM, due to a massive literature review, as summarized in Table 1. We apologize in advance for any omission, due to inadequacies in our literature survey.

### 2.1. Imidazolium-Based QA Head Groups of AEM

**Figure 4:**
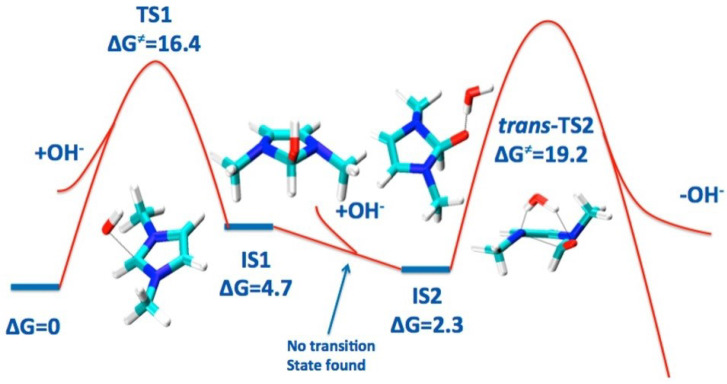
Free energy diagram for the nucleophilic addition–elimination pathway for the benzimidazolium-based head group (unit: kcal/mol). Reprinted, with permission from [110]. Copyright 2014, for the American Chemical Society.

DFT calculation for the imidazolium-based head group of AEM, in the presence of ${\mathrm{OH}}^{-}$ ion and implicit water molecules, was performed via implementing B3LYP, the polarizable continuum model (PCM), using Gaussian09 software. The effect of C2-substitution on alkaline stability and the degradation reactions of the imidazolium-based head group of AEM were studied [25,108,110,111]. Due to transition-state calculation for deprotonation and ring-opening reaction, it was found that the deprotonation of the imidazolium-based head group by the C2 atom occurs before the ring-opening reaction [25,108]. The dominant degradation mechanism of imidazolium-based and benzimidazolium-based head groups is a nucleophilic addition–elimination pathway, at the C-2 atom position on the imidazolium ring, as can be seen in Figure 4 [110,111]. On the other hand, the degradation reaction of the guanidimidazolium head group is followed by the ${\mathrm{OH}}^{-}$ ion attacking the guanidium part of the guanidimidazolium-based head group [109].

The transition-state-energy calculations for the dehydrogenation reaction, between various C2-substituted imidazolium and ${\mathrm{OH}}^{-}$ ions, by DFT, indicated the following order of alkaline stability: EMIIM (methyl) > EIIIM (isopropyl) > EPhIIM (phenyl) > EIIM (C2-insubstitued). EMIIM is the most stable one because EMIIM can hinder the deprotonation reaction most effectively, among the C2-substituted imidazolium head group of AEM, due to the highest electron-density distribution at beta-C [25,108].

The benzimidazolium-based head group degrades much faster than the imidazolium-based head group, due to the larger conjugation in its system. While the degradation mechanism of the substituted trimethylammonium head group depends on Hofmann elimination, the more stable cations are designed by increasing the alkyl chain [110,111]. A long alkyl chain will increase the steric effect, creating a Hoffmann elimination barrier, while the second way is to substitute alpha and beta hydrogens with other functional groups.

At the same time, the alpha carbon-methyl-substituted imidazolium cation (TMIM) was more stable than the alpha carbon unsubstituted imidazole head group (DMIM, BeMIM, BMIM), due to the hyperconjugation between the methyl group at the alpha carbon and the imidazole ring, as well as the steric effect of the methyl group [116]. The methyl-substituted imidazolium head group has more stability under alkaline conditions [116].

### 2.2. Alkylammonium-Based QA Head Groups

The degradation mechanism of substituted phenyltrimethylammonium head group in alkaline conditions was studied at the B3LYP 6-311G (2d,p) level with the polarizable continuum solvation model (PCM) in water [112]. Several substituents and their positions on the benzene ring were changed, in order to explore the relation between the orientation effect and the stability of the substituted phenyltrimethylammonium cations. The results of the DFT calculations indicated that the calculated energy barriers are raised, when the electron-donating substituents are at the ortho and para positions of the benzene ring. Specifically, the calculations showed that the double-${\left(\mathrm{CH}3\right)}_{2}$N-substituted phenylTMA+ is more stable than the double-${\left(\mathrm{CH}3\right)}_{2}$N-substituted benzylTMA+. These results elucidate the effects of substituents on the degradation of model cations and provide a reference for their potential use in anion-exchange membranes [112].

DFT calculations for the benzyltrimethylammonium-based AEM showed that as the water content reduced, the QA cations were degrading in the presence of ${\mathrm{OH}}^{-}$ at room temperature. However, with an increasing number of water molecules solvating the ${\mathrm{OH}}^{-}$, its nucleophilicity and basicity are hindering, and the QA degradation is significantly slowed [113].

DFT calculations for the tetraalkulammonium head group yielded that the nucleophilic substitution (second-order) and ylide formation, followed by Stevens and Sommelet–Hauser rearrangement, were the main degradation pathways [114,115].

### 2.3. Other Types of QA Head Groups

**Figure 5:**
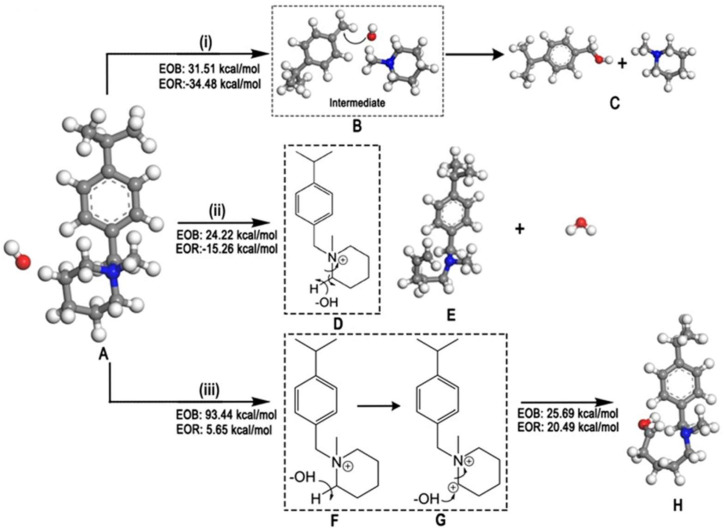
Degradation mechanism of N-methylpiperidine (NMP)-based AEM: (**i**) NMP head group detachment, (**ii**) ring-opening with subsequent formation of alkene, and (**iii**) ring-opening via direct hydroxylation. Reprinted with permission from [117]. Copyright 2020, for John Wiley and Sons.

The degradation mechanism of the vinyl benzyl head group. in the presence of implicit solvation and ${\mathrm{OH}}^{-}$ ions, were studied via the GGA-BLYP COSMO model and DMol [117]. As a result, it was found that the stability order of head groups is as follows: DABCO < TMA < NMP < ABCO. Furthermore, there is a multistep AEM degradation mechanism via the detachment of the whole vinyl benzyl head group. The first step is nucleophilic attack, leading to the loss of aromaticity, with subsequent transformation to a quinodimethane moiety. The second step is the detachment of the quinodimethane-like intermediate from the polymer backbone, by attacking superoxide or peroxy radicals via oxidative cleavage. The final step is the rearomatization of the reaction intermediate, as shown in Figure 5 [117].

The transportation of ${\mathrm{OH}}^{-}$ ions via QA functionalized polystyrene (QPS) AEM, in the presence of ${\mathrm{OH}}^{-}$ ions, implicit water molecules were studied with B3LYP 6-311 ++G(d,p) and the PCM model [50]. Two steps for ${\mathrm{OH}}^{-}$, transferring through QAPS-AEM were found. The first step was the movement of ${\mathrm{OH}}^{-}$ in the water channel, which was inducing by frequently forming and breaking hydrogen bonds (H-bonds) between $H_{2}O$ and ${\mathrm{OH}}^{-}$ [50]. The second step was that ${\mathrm{OH}}^{-}$ transferred across the QA head groups, by following the rotation of the C-C single bond, which was the rate-determining step for ${\mathrm{OH}}^{-}$, transferring in QAPS-AEM [50].

More recently, Karibayev et al. studied the chemical stability and transportation of ${\mathrm{OH}}^{-}$ ion trends, for the various QA head groups using quantum chemical properties, such as binding energies, LUMO energies, nucleophilic substitution reaction, and activation energies [94]. The results suggested that the trimethylhexylammonium-based QA head group is the most stable QA head group, while the pyridinium-based QA head group is the least stable QA head group.

To sum up, a number of DFT studies have been carried out to study degradation reactions of the various QA head groups of AEM. The DFT-based electronic structure modeling method is suitable for structure optimization and for exploring the mechanisms behind binding interactions and degradation reactions in detail.

## 3. Ab Initio Molecular Dynamics

QM/molecular mechanics (MM)-based ab initio MD is another powerful technique [118,119,120,121]. An ab initio MD calculation obtains trajectories of finite-temperature dynamics by implementing forces generated from ab initio calculations as the MD simulation proceeds. In addition, ab initio MD enables the chemical bond formation and breaking events as well as accounts for electronic polarization effects [118,119,120,121,122,123,124]. While both DFT and ab initio MD simulations are considered as ab initio calculations, DFT is a static method, while ab initio MD, also, describes dynamics, which is an important component when exploring both chemical stability and ${\mathrm{OH}}^{-}$ ion transportation. There have been several computational studies on chemical stability and ${\mathrm{OH}}^{-}$ ion diffusion mechanisms, under high and low HL of AEM, conducted via the ab initio MD method. The following paragraphs will illustrate a few recent ab initio MD studies on AEMs, as shown in Table 2. We apologize in advance for any omission, due to inadequacies in our literature survey.

### 3.1. Trimethylammonium-Based Poly(styrene)

The quaternized polystyrene-block-poly(ethylene ran butylene) block polystyrene AEM, in the presence of ${\mathrm{OH}}^{-}$ ion and water, were modeled and simulated with Quantum Espresso Package to study ${\mathrm{OH}}^{-}$ ion transportation [124]. Then, their findings are: the ion transportation depends on HLs and the location of ${\mathrm{OH}}^{-}$ ions in the polymeric system. The ${\mathrm{OH}}^{-}$ ions located in the dry zone or with a high coordination number are molecules with the lowest number of structural-diffusion events that effectively contribute to ${\mathrm{OH}}^{-}$ displacement. The results yielded that the Grottuss mechanism is dominant in its hydrated state and made several statements [124]. First, ${\mathrm{OH}}^{-}$ ions partially dissociate, due to donor-acceptor interactions acting competitively on them, when HL is 4. Second, dissociation of ${\mathrm{OH}}^{-}$ ions, completed at water uptake 6, and ${\mathrm{OH}}^{-}$ ions conform to hypercoordinated structure, similar to the square-planar arrangement, described for pure water medium [124].

### 3.2. Trimethylammonium-Based Graphene Bilayer

The structural properties, ${\mathrm{OH}}^{-}$ ion solvation, and transportation pattern in each water layer of carbon nanotubes or graphene-bilayer-based AEM, at the higher HL, were studied via the ab initio MD simulation [40,42]. The results of the simulations illustrated that the various nanoconfined water structures play an important role in understanding the ${\mathrm{OH}}^{-}$ solvation pattern and ${\mathrm{OH}}^{-}$-ion-transportation mechanism. The nanoconfined water structures were significantly changed, by varying the HL, cation spacing, AEM width, and geometry [40]. The various ${\mathrm{OH}}^{-}$-ion-coordination patterns and solvation complexes were noted, in each water layer. The various water layers could suppress or promote the transportation of ${\mathrm{OH}}^{-}$ ions, in AEMs.

**Figure 6:**
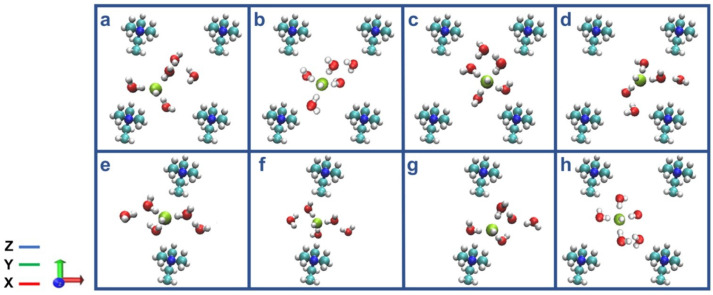
The detailed z-axis illustration of vehicular diffusion mechanism steps (**a**–**h**). Representative configurations showing the vehicular diffusion for the simulated system from a z-axis perspective, in the center of the cell (**a**–**d**) and in the bottleneck region (**e**–**h**), including five water molecules from the first and second solvation shells. Red, white, turquoise, and blue spheres represent O, H, C, and N atoms, respectively. A green sphere represents the hydroxide ion. (**a**) A hydroxide ion is in a stable threefold structure near a cation, with two water molecules in the second solvation shell. (**b**) The hydroxide ion is in a fourfold planar structure in the center of the cell, with one water molecule in the second solvation shell. (**c**) The hydroxide ion and the five water molecules move toward the nearby cation. (**d**) The hydroxide ion is in a stable threefold structure near a cation. Two water molecules are located at neighboring bottleneck regions. (**e**) The hydroxide ion forms a stable ${\mathrm{OH}}^{–}{\left(H_{2}O\right)}_{4}$ complex, in which three water molecules are part of a threefold tetrahedral structure, and one water molecule is in the second solvation shell. A water molecule is located below the hydroxide ion in the bottleneck region. (**f**) The hydroxide ion and five water molecules are diffusing through the bottleneck region. (**g**) The hydroxide ion crosses the bottleneck region and is located near a cation in a stable threefold structure. (**h**) The hydroxide ion’s threefold structure changes back into a fourfold planar structure, as it diffuses toward the center of the cell. Reprinted with permission from [41]. Copyright 2019, for the American Chemical Society.

Moreover, the ${\mathrm{OH}}^{-}$ ion solvation and transportation in confined to the graphene bilayers of AEM at the lower HL were, also, studied in details [41]. The six different idealized distribution of water models in AEM were created based on the various hydration degree and the spacing head group within the AEM. The research outcome argued that the various water distribution in AEM is a critically important descriptor, compared to the value of HL for the classification of AEMs’s working principle at the lower HL. In addition, the various ${\mathrm{OH}}^{-}$ ion transportation mechanisms depend on the absence or presence of a second solvation shell of the ${\mathrm{OH}}^{-}$ ion and on the local water structure [41]. The vehicular diffusion in AEM was, also, described in detail, as shown in Figure 6.

From Figure 6a, it can be noted that the ${\mathrm{OH}}^{-}$ ion is in a stable threefold structure, near a head group, and has two water molecules in the second solvation shell. Next, the ${\mathrm{OH}}^{-}$ ion is changed into a fourfold planar form, via having one water molecule in the second solvation shell, as shown in Figure 6b. After that, the five water molecules and one hydroxide ion transport, toward the head group, as shown in Figure 6c. Then, ${\mathrm{OH}}^{-}$ ion forms a stable threefold structure, near a head group, as shown in Figure 6d. A stable ${\mathrm{OH}}^{-}{\left(H_{2}O\right)}_{4}$ complex was formed, and then one water molecule was present in the second solvation shell, as shown in Figure 6e. The diffusion of ${\mathrm{OH}}^{-}$ ion and five water molecules via the bottleneck region are illustrated in Figure 6f. Then, ${\mathrm{OH}}^{-}$ ion crossed the bottleneck region of AEM and was located near a head group, in a stable threefold structure, as shown in Figure 6g. In addition, finally, the threefold geometry of ${\mathrm{OH}}^{-}$ ion changed back to a fourfold planar geometry, as it diffuses toward the center of the cell, as shown in Figure 6h [41].

**Figure 7:**
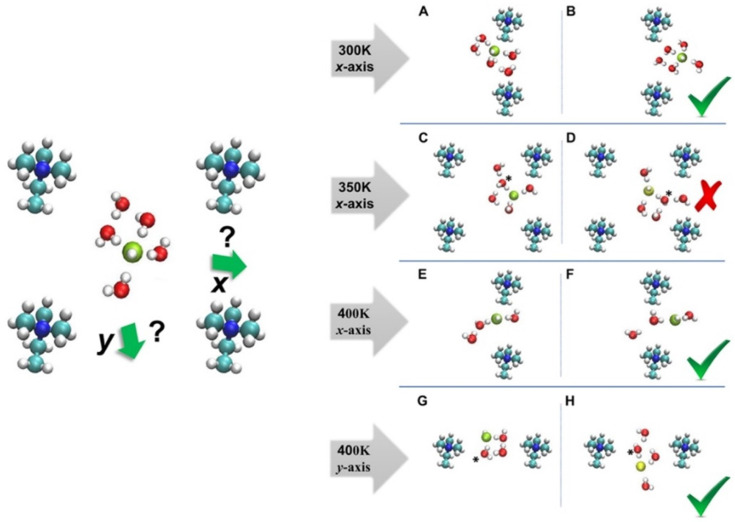
The various configurations for designed distribution of water in AEM at the different axis. Green and yellow spheres represent the initial and final hydroxide ion oxygen atoms, respectively. (**A**,**B**) ${\mathrm{OH}}^{-}$ exhibits vehicular diffusion at room temperature, forming a stable ${\mathrm{OH}}^{-}{\left(H_{2}O\right)}_{5}$ complex, in which three water molecules are part of a threefold tetrahedral structure (with one water molecule located below the hydroxide in the bottleneck region, while two water molecules are in the second solvation shell. (**C**,**D**) The hydroxide ion is trapped in the center of the cell at 350 K, as a result of a proton rattling event. (**E**,**F**) ${\mathrm{OH}}^{-}$ exhibits vehicular diffusion along the x-axis at 400 K, forming a twofold structure, as a result of the increased mobility of the water molecules. (**G**,**H**) ${\mathrm{OH}}^{-}$ exhibits diffusion along the y-axis at 400 K, due to the high mobility of the water molecules. The hydroxide ion diffuses via vehicular diffusion toward the bottleneck region; a proton-transfer event, then, occurs in the bottleneck region, which places the nascent hydroxide into the center of the cell. Reprinted with permission from [43]. Copyright 2022, for the American Chemical Society.

The effect of temperature on the diffusion of ${\mathrm{OH}}^{-}$ ions in the confined graphene bilayer of AEMFC was, also, explored via the ab initio MD [43]. The results stated that the ${\mathrm{OH}}^{-}$ ion diffusion changes non-monotonically, by raising the temperature. Namely, “diffusion kink” was present in the temperature versus diffusivity curve at the different HLs. Therefore, it was expected for the discovery of this “kink” to play a crucially important role in the design of highly chemically stable and improved transportation of ${\mathrm{OH}}^{-}$ ion for AEMFCs [43]. The various snapshots of configurations for the nonuniform water distribution in AEM were illustrated in Figure 7.

The results of the above study claimed that the highest ${\mathrm{OH}}^{-}$ ion and water diffusion coefficients are obtained at 400 K, for the system illustrated in Figure 7. The vehicular diffusion of ${\mathrm{OH}}^{-}$ ion at room temperature was observed via the formation of ${\mathrm{OH}}^{-}{\left(H_{2}O\right)}_{5}$ complex, as shown in Figure 7A,B. The ${\mathrm{OH}}^{-}$ ion trapped in the center of the AEM was due to the proton-rattling event, as shown in Figure 7C,D. The analysis of ab initio MD results, also, claimed that the highest ${\mathrm{OH}}^{-}$ ion diffusion and water diffusion coefficient were obtained at the 400 K. For instance, the vehicular diffusion of ${\mathrm{OH}}^{-}$ ion was reported for the x-axis at 400 K, as shown in Figure 7E,F. In addition, ${\mathrm{OH}}^{-}$ ion exhibited a diffusion event in y-axis at 400 K, due to the high mobility of water, as shown in Figure 7G,H.

In summary, several studies have been performed to investigate the poly(styrene) and graphene-bilayer-based AEM structure via ab initio MD simulations. The ab initio MD simulations enabled us to study (i) the structural properties of AEM, (ii) the mobility of the ${\mathrm{OH}}^{-}$ ion, and (iii) the structural diffusion of the ${\mathrm{OH}}^{-}$ ion in AEM, including vehicular and Grottus mechanisms, to name a few.

## 4. All-Atom Molecular Dynamics (MD) Simulations

In the last decades, the technological advances in computational physics, chemistry, and other fields have pushed the scientific community forward, to study the dynamics of complex systems at the atomic resolution. In MD simulations, the total forces on all the atoms are calculated, and then the dynamics of designed systems are simulated by discrete integration of Newton’s equations of motions with a shorter timescale, to determine the movement atom’s response to those forces [125,126,127,128]. There is a wide range of MD simulations: rational design and development of drugs, catalysts, conformational analysis of proteins, polymers, the molecular formation mechanism of green solvents, aerosols, transportation of lithium-ion in electrode, transportation of ${\mathrm{OH}}^{-}$ ion in the cationic-head-group-based AEM, and others [120,129,130,131,132,133,134,135,136,137,138,139,140]. The standard software for MD simulations includes, to name a few, in alphabetical order: AMBER SUITE, CP2K, GROMACS, NAMD, LAMMPS, and others [139,140,141,142,143,144,145,146,147,148,149,150,151].

The timescale of rare events needs more time in classical all-atom MD, resulting in the development of enhanced sampling methods. More simulation time is spent to sample rare events with a high-energy state [150]. High-energy barriers separate the different metastable states, and transitions between those states are called rare events, which occur on a longer timescale [151]. During the last decades, different methods with improved, enhanced sampling for all essential regions of free energy landscape have developed, to alleviate this timescale problem. The enhanced sampling methods are umbrella sampling, the free energy perturbative approach, thermodynamic integration, conformational flooding, adiabatic MD, local elevation methods, steered MD, adaptive force bias, Jarzynski’s identity-based approach, weighted histogram method, metadynamics, and others [152]. There are many computational methods to study ion-ligand binding free energy. For instance, Li and Merz Jr. reviewed the binding model of ion-ligands, such as unpolarizable, polarizable, angular overlap, and valence-bond-based, using quantum and classical mechanics approaches [153]. It is out of the scope of this review to go through all the mentioned different methods. Therefore, it is essential to note that all different free energy calculation methods have points of strength and limitations, and no methods outperform all others.

**Figure 8:**
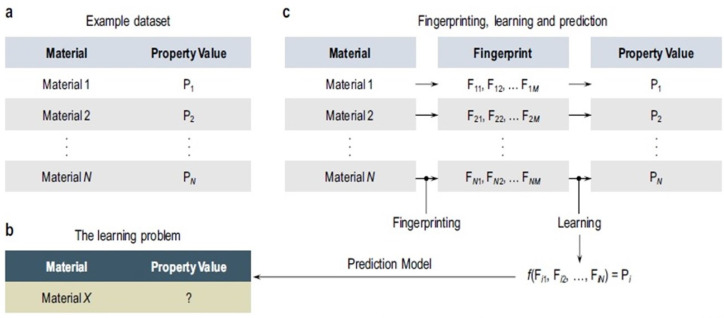
Key elements of machine learning in materials science. (**a**) Schematic view of an example dataset, (**b**) statement of the learning problem, and (**c**) creation of a surrogate prediction model, via the fingerprinting and learning steps. *N* and *M* are, respectively, the number of training examples and the number of fingerprint (or descriptor or feature) components. Reprinted with permission from [162]. Copyright 2017, for Springer Nature.

In recent years, many researchers studied the development and applications of machine learning (ML) models in molecular design and performance improvement of energy materials and devices [154,155,156,157,158,159]. ML trained, based on QM/MM, may allow MD simulations at an accurate level close to the electronic-structure method chosen to generate a training set [160,161]. The implementation of ML-based methods enable researchers to predict the material properties, based on databases already obtained from DFT, MD, and other simulation methods. Careful selections of feature values are required for the material property prediction by ML. The artificial neural network is considered a subcategory of ML and is, also, integrated with DFT, MD, and other simulation methods [162,163]. The workflow of ML algorithms in material science is illustrated in Figure 8 [162]. Essentially, the existence of past data is a prerequisite for ML, as shown in Figure 8a. Then, we define the target material (‘Material X’), with the unknown property, as an ML problem in Figure 8b. At the same time, we predict that unknown property by fingerprinting and a learning algorithm, shown in Figure 8c [162].

Many MD studies were devoted to studying the transportation mechanism of ${\mathrm{OH}}^{-}$ ions, from various matrices of AEM, to increase IEC. Many types of polymers have been investigated computationally, as a backbone for the matrix of AEMFCs, including poly(p-phenylene oxide) (PPO), poly(vinyl benzyl) (PVB), poly(arylene ether sulfone ketone), poly(ether ether ketone) (PEEK), polysulfone (PS), and norbornene [164]. Those polymers have good mechanical processability, improved chemical and mechanical stability, and low cost, plus are easily functionalized with cationic-functional-head groups [164,165]. There have been a number of computational studies on the transportation mechanism of ${\mathrm{OH}}^{-}$ ions, in AEMs applying MD simulations. The following paragraphs illustrate the results of a massive literature review about various MD modeling and simulations of AEMs, as can be seen in Table 3. We apologize in advance for any omission due to inadequacies in our literature survey.

### 4.1. Varous QA Head Groups of AEM

The classical all-atom MD simulations for different QA head groups were performed in the presence of SPC/E water model, ${\mathrm{OH}}^{-}$ ion, with the OPLS force field and LAMMPS software to study the critical relationship between the chemical stability of the QA head group in AEMFC environments [166]. The results of classical all-atom MD simulations stated that (i) QA head groups are unstable at low HL, as QAs rapidly degrade, which in line with experimental findings, and (ii) high temperature, also, increases the degradation of QAs at low HL [166].

### 4.2. Functionalized Poly(phenylene Oxide) Based AEM

PPO is an amorphous and high-temperature thermoplastic discovered by Allan Hay and commercialized by General Electric in 1960. PPO has many applications, such as electrolytes for electronics, household, lithium-ion batteries, and AEM backbone. Li and coauthors suggested that PPO-based AEM’s mechanical instability caused by chain scission after exposure to the alkaline solution [176]. Parrondo and their research group suggested that the electron-withdrawing effect of a tethered QA head group is triggering PPO-based AEM degradation [165]. At this time, many MD studies were studying those PPO-based AEM membranes.

From the viewpoint of the molecular level, Zhang et al. performed classical all-atom MD simulations for an imidazolium-group-grafted PPO chain, under the presence of ${\mathrm{OH}}^{-}$ ions and water molecules as an explicit solvent, with a CHARMM force field and NAMD software to examine the hydration of imidazolium group and polymer structure [167]. The findings illustrated that the desired balance between the affinity of imidazolium group to ${\mathrm{OH}}^{-}$ ion and transportation of ${\mathrm{OH}}^{-}$ ion in hydrated imidazolium group-grafted PPO chain-based AEM was achieved, under critical water-saturation conditions, which means 2/8 water molecules were present in the first/second hydration shell [167].

In addition, the imidazolium-based head group structure’s effect on ${\mathrm{OH}}^{-}$ ion diffusion and chemical stability of AEM were, also, investigated by classical all-atom MD simulation using Materials Studio, COMPASS II Force field [140]. The results of classical all-atom MD simulations with COMPASS II Force field yielded that the PPO AEM with 1,2,4,5-tetramethylimidazolium and alkyl spacer chain with six or eight aliphatic carbons at the HL 6 illustrated an excellent balance between chemical stability and ${\mathrm{OH}}^{-}$ ion diffusivity for AEM. The outcome of this work, also, provides a good design principle for the imidazolium head group-based PPO backbone of the AEMFC application.

**Figure 9:**
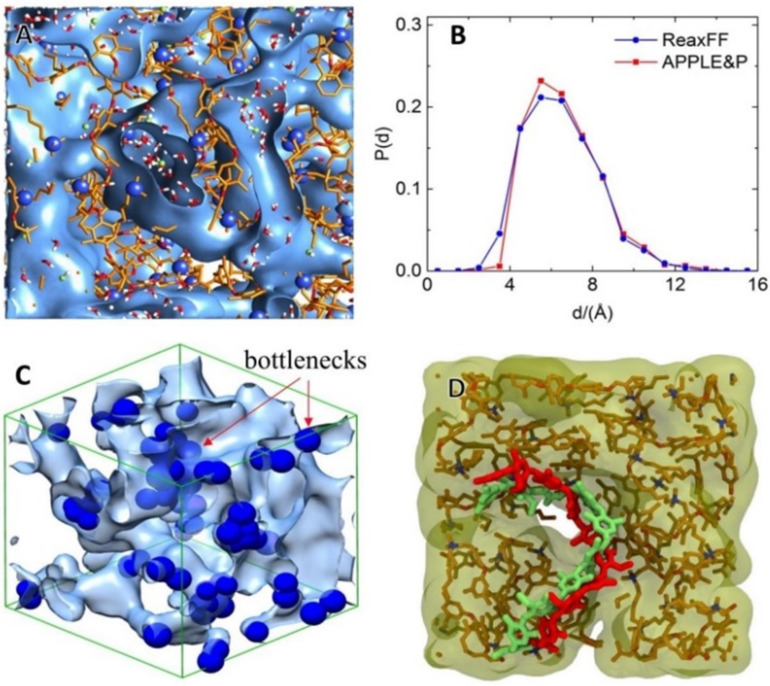
(**A**) Snapshot of hydrated M1 membrane with water channels illustrated by isosurfaces, corresponding to 50% of bulk water density. (**B**) Distribution of water channel width. (**C**) Blue spheres illustrate the locations of the “bottlenecks” inside water channels. (**D**) Illustration of correspondence of membrane morphologies obtained from the APPLE&P simulation and mapped to ReaxFF. The red chain shows a polymer backbone for the selected chain in the APPLE&P simulation, while the green chain shows the same chain after mapping and relaxation in ReaxFF simulation. Reprinted with permissions from [36]. Copyright 2020, for the American Chemical Society.

The various head-group-based PPO AEM including trimethylamine, dimethyl butylamine, dimethyl octylamine, dimethyl hexylamine, tripropylamine, and dimethyl methoxy butyl amine hydrated with ${\mathrm{OH}}^{-}$ ion and water molecules were, also, modeled and studied via the implementation of ReaxFF (reactive) and APPLE&P (non-reactive) classical polarizable force fields [168,169,177]. The diffusion of ${\mathrm{OH}}^{-}$ ion is improved by forming a water channel under high water content (Figure 9). Improved alkaline stability was achieved, by replacing the methyl group of the QA head group with larger hydrophobic groups, which block the ${\mathrm{OH}}^{-}$ ion approaching toward nitrogen of QA. The loss of coordinated water molecules from ${\mathrm{OH}}^{-}$ ion, observed during the vehicular transport mechanism (non-reactive MD) through bottlenecks in the water channel, creates a more significant kinetic barrier for such an event. As was, also, found, the Grotthuss mechanism is essential for understanding ${\mathrm{OH}}^{-}$-ion diffusion, by water channels in non-blocky polymer-structure-based AEMs, and ${\mathrm{OH}}^{-}$ ions can be transported without damage or loss of the ${\mathrm{OH}}^{-}$-ion-hydration structure or loss of its coordinated water molecules, with a lower transition barrier (the bottleneck is an easy transportation pathway) [36,168,169,177].

### 4.3. Functionalized Poly(vinyl)-Based AEM

Polyvinyl chloride is the most popular plastic polymer and has a wide range of applications in the production of doors, windows, food packing, etc. There are many derivatives of polyvinyl chlorides, such as polyvinyl alcohol, polyvinyl benzyl, and others. The poly(vinyl benzyl) trimethylammonium has a stable environment in alkaline solution, due to good anionic conductivity and the absence of beta hydrogen atoms. A few recent MD simulation studies have investigated the vehicular and Grottuss mechanism of PVB polymer in alkaline media [178,179].

Regardless, the four types of PVB polymer chains in the presence of SPC/Fw water model, ${\mathrm{Cl}}^{-}$ and $F^{-}$ ions were modeled and simulated, using the AMBER force field and the LAMMPS package, in order to illustrate a novel “co-ion effect” where ${\mathrm{Cl}}^{-}$ ion could significantly improve $F^{-}$ ion transportation in the AEM [170]. The results of classical all-atom MD simulations concluded that the enhancement in $F^{-}$ ion mobility, as ${\mathrm{Cl}}^{-}$ ion content increases from 0% to 90%, is due to the larger size of the ${\mathrm{Cl}}^{-}$ ion, which more readily loses its water solvation shells because of a lower charge/radius squared. In addition, there was a debate about Grotthuss hopping and the vehicular diffusion mechanism of ${\mathrm{OH}}^{-}$ ions by AEM, which motivated many scientists to study this phenomenon for the PVB system [170].

At the same time, Chen et al. used classical all-atom MD simulations for the PVB (10, and 40 monomers) in the presence of ${\mathrm{OH}}^{-}$ ions and aSPC/Fw water model, via the general AMBER force field and LAMMPS software [32]. The ${\mathrm{OH}}^{-}$ ion transportation is, primarily, based on vehicular diffusion (80%), with the remaining 20% based on Grotthuss diffusion [32].

Meanwhile, Dubey et al., also, performed classical all-atom MD simulations for 40 monomers of 1 PVB polymer chain, with 120–720 water molecules, OPLSAA and SWM4-NDP force fields, to study the vehicular diffusion and solvation structure of ${\mathrm{OH}}^{-}$ ions in an AEM [39]. The authors state that the vehicular diffusion mechanism contributes 11.5% of the total diffusion of ${\mathrm{OH}}^{-}$ ion, which contradicts the findings of Chen et al. [39].

Hence, Chen et al. studied PVB and calculated the vehicular diffusion coefficient, by decomposing the total mean square displacement into discrete (hopping) and continuous (vehicular) ones [32]. In contrast, Zhang et al. studied the PPO with a ReaxFF/classical force field and calculated vehicular-diffusion, by switching off a reactive part [167,168]. Vehicular diffusion influenced by Grotthuss diffusion, during the decomposition method, could lead to a higher contribution of vehicular diffusion. On the other hand, the diffusion of water via the AEM matrix is 15 times slower than that in bulk water at the same temperature condition, due to the confinement effect by the AEM.

### 4.4. Functionalized Poly(arylene Ether Sulfone) Based AEM

The one chain of ethyl imidazolium-functionalized poly(arylene ether sulfone) with 31 ${\mathrm{OH}}^{-}$ ions and 295/622 water molecules were modeled and simulated using COMPASSII force field and Material Studio software to study the effect of functional group types on water channel morphology [171]. Ethyl imidazolium-functionalized poly(arylene ether sulfone) has a high ${\mathrm{OH}}^{-}$-ion conductivity because of well-defined phase separation morphology and chemical stability in comparison with QA functionalized one. The results implied that the ethyl imidazolium head groups based on AEM had more effective water channels and higher chemical stability than the QA-head-group-based AEMs, and conjugated p-bonds of heterocyclic systems enhance this chemical stability. In addition, ethyl imidazolium (a small functional group) had a lower ion conductivity than bulky functional groups, such as quaternary phosphonium and tertiary sulfonium at the same hydration conditions [171].

The classical all-atom models of QA-substituted fluorenyl group-based poly(arylene ether sulfone ketone)s (QPE), in the presence of water and ${\mathrm{OH}}^{-}$ ion, were simulated via CVFF forcefield to study the transportation of ${\mathrm{OH}}^{-}$ ions [172]. Then, it was found that microscopic hydrated-water structures around QPE and ${\mathrm{OH}}^{-}$ ion conductivity are independent of 10–20 repeating units. The outcomes, also, claim that the surface diffusion mechanism is a driving path for ${\mathrm{OH}}^{-}$ transportation. The radial distribution function results imply that half of ${\mathrm{OH}}^{-}$ ions were hydrated, while the other half interacted with QA head groups [172].

### 4.5. Functionalized Poly(Ether Ether Ketone)-Based AEM

The classical all-atom MD models for PEEK (10–40 units), in the presence of ${\mathrm{OH}}^{-}$ ions and water, were simulated via COMPASS force field and Material Studio software, to study the effect of the QA head groups, of polymer, on hydronium and ${\mathrm{OH}}^{-}$ ion transportation [173]. The results showed that the ${\mathrm{OH}}^{-}$ and hydronium ions transportation in the PEEK membrane increased as the mole ratio of the functionalized moiety increased. At the same time, at a large water amount in the simulated cell, the polymer density of the functional group is reduced, due to the salvation effect of water, which leads to reduced density of polymer and poor connectivity of ionic sites [173].

### 4.6. Functionalized Poly(Sulfone)-Based AEM

Classical all-atom MD models for trimethyl ammonium PS-based AEM with ${\mathrm{OH}}^{-}$ and water (TIP5P) were simulated, using the DREIDING forcefield and Gromacs software, to study the effect of HL on diffusivity [174]. As a result, the AEM structures consist of hydrophilic domains connected through dynamic water nanochannels, where the percolation degree increases with IEC and water uptake. In addition, the trimethylammonium has fixed-charge groups along the polymeric chain and tends to interact to maximize H-bond and electrostatic interaction. At low IEC and water uptake conditions, the ${\mathrm{OH}}^{-}$ ion hydrated poorly, and the trimethylammonium donated 2–3 coordinating water molecules, to balance water distribution inside the membrane [174].

In addition, the PS-based AEM and proton exchange membranes in the presence of water, ${\mathrm{OH}}^{-}$ and $H^{+}$ ions were simulated using the DREIDING force field and the LAMMPS software to compare their nanophase-segregated structure and transport properties [51]. The ${\mathrm{OH}}^{-}$ ions and QA group of AEM are more solvated with water than the hydronium ion and sulfonate group of PEM. The better solvation of the QA head groups and ${\mathrm{OH}}^{-}$ ions yield less mature hydrogen bonding in its internal structure, especially at lower water-content conditions [51].

### 4.7. Functionalized Poly(Nonbornene)-Based AEM

The poly(norbornene) polymer chains, in the presence of water and ${\mathrm{OH}}^{-}$ ions, with models simulated using Materials Studio software, to examine the interaction between polymer chain and water molecules as well as transportation of ${\mathrm{OH}}^{-}$ ions [175]. The results showed that (i) the QA head groups were evenly distributed around the water channels, (ii) ${\mathrm{OH}}^{-}$ ions surrounded by and migrated between two layers of water shells, and (iii) ${\mathrm{OH}}^{-}$ ions moved faster with increasing temperature, due to the higher kinetic energy of ${\mathrm{OH}}^{-}$ ions [175].

The coordination number between the poly(norbornene) polymer chain and ${\mathrm{OH}}^{-}$ ions, and the transportation mechanism of ${\mathrm{OH}}^{-}$ ion were, also, studied by the classical all-atom MD simulations [90]. It was found that (i) diffusivity of ${\mathrm{OH}}^{-}$ ion increased with increasing the temperature due to the higher kinetic energy of ${\mathrm{OH}}^{-}$ ions and (ii) diffusivity of ${\mathrm{OH}}^{-}$ ion increased with increasing water content and channel size, due to the favoring of continuous water channels, combining the Grotthuss mechanism and the vehicle mechanism [90].

A various computational works were performed to investigate the poly(phenylene oxide), poly(vinyl), poly(arylene ether sulfone), poly(ether ether ketone), poly(sulfone) and poly(norbornene)-based AEM at the molecular level. The classical all-atom MD simulations were, mainly, enabled us to study (i) nano-phase-segregated water channel and polymeric-backbone structures of AEM, (ii) calculate the ionic conductivity based on diffusion coefficient using Nernst–Einstein equations, and (iii) the ${\mathrm{OH}}^{-}$-ion-diffusion coefficient for Grottus and vehicular transportation mechanisms. However, classical all-atom MD is not appropriate for the simulation of chemical degradation reactions and transportation of ${\mathrm{OH}}^{-}$ ions, as they cannot capture the creating and breaking of chemical bonds. The rapid development of the QM/MM technique (ab initio MD) can simulate chemical degradation reactions and transportation of ${\mathrm{OH}}^{-}$ ions. Therefore, ab initio MD is an optimal method for exploring such reactions. However, the ab initio MD method, typically, has a high computational cost.

## 5. Coarse-Grained Molecular Dynamics Simulations

CGMD modeling aims to simulate complex systems’s behavior via coarse-grained representation [180,181]. The coarse-grained model reduces the number of degrees of freedom in a system, by reducing the number of interaction sites, resulting in a computationally less expensive model than the equivalent fully atomistic model [181,182,183,184]. One of the methods to develop effective interactions at this mesoscale level is the so-called “iterative Boltzmann inversion” method. In this method, a numerical non-bonded potential updated iteratively until the trial radial distribution functions match within some tolerance, and secondly, the target data obtained from the atomistic simulations are mapped to the CG level [184,185,186,187,188,189,190,191].

Typically, each bead represents three to five heavy atoms and their pendant hydrogen atoms [190,191,192]. This kind of mapping aims to replace functional groups with corresponding beads that represent their level of polarity and affinities to other chemical groups. Specific interactions, i.e., hydrogen bonds, do not, explicitly, model. The advantage of this approach is that it is a straightforward extension of the tools used in atomistic MD. Bead interactions are modeled via the effective coarse-grained potentials. Bead motion is simulated with the same Newtonian dynamics, but with a time step that can be orders of magnitude larger [190,191,192,193,194]. Table 4 summarizes a few selected CGMD studies on AEMs. We apologize in advance for any omission due to inadequacies in our literature survey.

### 5.1. Functionalized Poly(phenylene Oxide)-Based AEM

**Figure 10:**
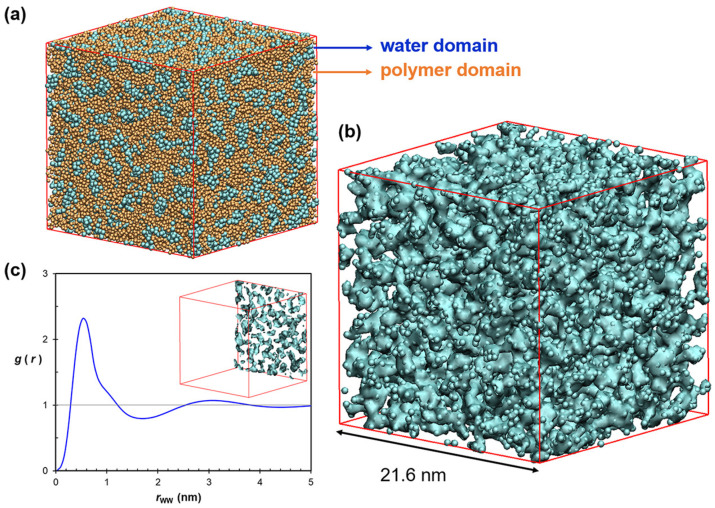
(**a**) Bead-based visualization of a simulated PPO-Q system (degree of functionality: 20%, water uptake: 20 wt%, ion exchange capacity: 1.48). Cyan beads are water beads (type W), and orange beads are polymer beads (type B). (**b**) Isosurface around the W beads visually illustrating the water domain. (**c**) Radial distribution function of W beads; the location at which the first peak drops below 1 is 1.2 nm. The inset shows the isosurface of W beads in the clipped bottom of the simulation box. Reprinted with permissions from [177]. Copyright 2020, for the American Chemical Society.

A dissipative particle dynamics (DPD) simulation was applied, to study the nanostructure and ion diffusivity of PPO matrix-based AEM, via various process parameters, including alkyl-chain length, side-chain structure, and side-chain distribution, as shown in Figure 10 [177].

As a result, modifiers of the alkyl-side-chain achieve nanosegregation of hydrophilic and hydrophobic domains. In addition, the ionic pathways formed in lamellar structure, when side chains are distributed normally, act as comb-like structures and reach an ionic conductivity of 17 mS/cm [177].

The ion transportation pathway id explained by studying the effect of hydrated PPO structure on HLs and IECs in spacers. [196]. The results implied that the diffusivities of water and anions increase with an elevation of HL and IEC. In addition, there was the formation of larger water clusters and nanophase segregation during the presence of alkyl spacers. Additionally, the cluster size is increasing further because of the agglomeration with increasing HL or length of alkyl spacers [196].

Lu et al. developed the high-resolution CG MD model for PPO-based AEMFC in the presence of explicit water models with Martini FF [197]. The authors claimed that this model applied for desalination, water purification, and redox flow batteries [197].

The study of the effect of water content and ionomer architecture on the nanostructure and ion conductivity of AEM, based on PPO by Lu et al., implies that the ion conductivity of the AEM is very sensitive to water content but less sensitive to changes in the architecture of the polymer matrix of AEM via CGMD [195]. Therefore, it is suggested that the ionic conductivity of AEM improved via the relationship between polymer chemistry and equilibrium water uptake [195].

### 5.2. Functionalized Poly(ether Ether Ketone)-Based AEM

The two different PEEK-based membranes, which include one side chain (SQ) and two side chains containing (QA) (GQ), were studied via the CGMD model by Martini FF [198]. First, the results reported that the self-diffusion coefficients are similar for SQ and GQ, meaning they have a similar IEC. More water molecules wrap around the ${\mathrm{OH}}^{-}$ ions in GQ, which could improve alkaline stability compared to SQ [198].

### 5.3. Functionalized Poly(styrene-b-poly(ethylene-co-butylene)-b-polystyrene-Based AEM

The DPD simulation for polystyrene-b-poly(ethylene-co-butylene)-b-polystyrene (SEBS), in the presence of water and ${\mathrm{OH}}^{-}$ ions, was performed to study the hydrated morphology and microstructure of an alkyl-substituted ionomer [52]. The outcome revealed that (i) domains exclusively consisting of water were generated at high HL, within the hydrophilic phase, (ii) ${\mathrm{OH}}^{-}$ ion is three-fold coordinated or less at low degrees of HL and cannot be well represented with four-fold DPD beads, and (iii) larger exclusive domains of water were formed at the highest HL when changing ${\mathrm{OH}}^{-}$ ions to the ${\mathrm{Cl}}^{-}$ ions [52].

The morphology of various SEBS-based ionomers: SEBS trimethylammonium, SEBS—methylimidazolium, SEBS—trimethylphosphonium was studied as a function of the HL in detail [200]. This CGMD study illustrated that the morphology was transformed to perfect lamellae, followed by disordered bicontinuous domains from perforated and interconnected lamellae, as the HL was increased from 4 to 20 lambda. The distribution of water did not change, but the backbone structure changed less during the selection of the functional group [200].

In addition, the effect of alkyl spacer on the hydrated morphology of AEM was, also, studied via DPD simulations [199]. The results claimed that HL number 12 was most likely to form lamella structure for SEBS-TMA, -TMPA, -MDPA, and -DMPA-based AEMs. The alkyl linker created flexibility in the side chain, leading to an extension of the backbone TMA distance and uniformity of its distribution. Cluster analysis illustrated that the percolation formed at HL 8 for SEBS-TMA, -TMPA, and -DMPA, but HL 12 for SEBS-MDPA, with more spacers per side chain [199].

A series of works were performed to study the PPO, PEEK, and SEBS-based AEM, at the coarse-grained scale. The current studies were essential to the rational design of the effect of polymer architecture, side-chain, hydrophobic and ionic interactions, and morphology on the various polymeric-backbone-based AEM. DPD modeling and simulation techniques were, mainly, implemented to investigate the various polymeric-backbone-based AEMs.

## 6. Continuum Modeling and Simulation

Continuum mechanics deals with the mechanical behavior of materials modeled as a continuous mass rather than as discrete particles [201,202,203]. Modeling an object as a continuum ignores that matter is made of atoms and is not continuous. However, such models are highly accurate on length scales much greater than inter-atomic distances [203,204,205]. Fundamental physics laws, such as the conservation of mass, the conservation of momentum, and the conservation of energy, are applied to such models to derive differential equations describing the behavior of such objects. Information (material properties) about the particular material added by constitutive relations [205,206,207].

**Figure 11:**
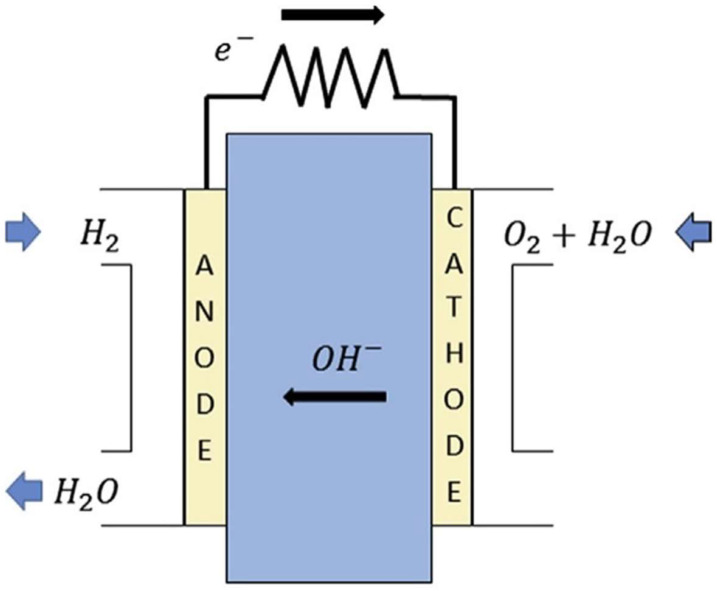
A schematic representation of AEMFC. Reprinted with permission from [53]. Copyright 2017, for Elsevier.

On the system level, a typical AEMFC consists of its constituting electrodes (cathode and anode) and MEA (GDL, CL, and AEM) parts. As an example of continuum-modeling studies of AEMFCs, Machado et al. [208,209,210] carried out several continuum-level modeling studies on AEMFCs. Firstly, the effect of process parameters, such as flow direction, temperature, and relative humidity were studied by this research team. Secondly, the authors investigated an agglomerate model and parametric study, using air at the cathode. Thirdly, the author studied the entropy generation analysis, based on a three-dimensional agglomerate model. Fourthly, the agglomeration model of 3D AEMFC using the finite-volume-modeling approach was explored, as shown in Figure 11. As a result, the authors came to several conclusions. Firstly, AEMFC performance improved by lowering the relative humidity of the cathode side and increasing the membrane water content, platinum loading, and ionomer volume fraction. Reversible and irreversible heat were identified as the primary sources of entropy production for all the parameters tested. Thirdly, the macro-homogeneous model overestimated the cell performance compared to the agglomerate model, due to the resistances associated with the species and ionic transports in the CL [208,209,210].

Dekel et al. [211,212] developed a one-dimensional model of an AEMFC, capable of predicting time-dependent performance and performance stability at high current densities. The model relates ionomer degradation, hydration, and operating conditions. In a separate study [213], their modeling results have, clearly, shown that, while improved AEM hydroxide conductivity is truly important for the achievement of high cell performance, enhanced water diffusivity through the membrane is extremely critical to ensure long-term AEMFC performance stability, as required by practical automotive and other applications. Overall, their model has been demonstrated to be a useful tool for parameter sensitivity analysis, optimization studies, and cell design.

## 7. Summary and Outlook

This review presents recent modeling and simulation studies of the chemical degradation of the QA head groups and the transportation of ${\mathrm{OH}}^{-}$ ions at the different scale. Nowadays, many methods and software packages for molecular and materials modeling are available. Applications of such methods may help to understand the transportation mechanisms of ${\mathrm{OH}}^{-}$ ions, chemical stability of functional head groups, and many other relevant properties, leading to a performance-based molecular and structural design as well as, ultimately, improved AEM-based fuel-cell performances.

In QM-based DFT calculations, the investigated QA head groups of AEMs are, usually, a small fraction of the polymers in realistic experimental and industrial systems. At the same time, the process parameters, such as temperature, pressure, and hydration level, remain idealized, as compared to experimental realistic conditions. Many researchers implemented an implicit solvation model, for the study of AEM by DFT, and the implicit solvation model states that the solvent (water) does not interact with solutes, such as (${\mathrm{OH}}^{-}$ ions and the backbone of AEM based on the QA head groups). Due to those weaknesses, researchers in this area might not be convinced by DFT results. However, DFT models implement the chemical and physical laws of natural phenomena and can, in principle, provide us with an understanding of the chemical-degradation-reaction mechanisms of head groups of AEMs.

Ab initio, classical all-atom MD, and coarse-grained MD have all been actively applied to study AEM-related problems. Some authors, also, calculated ${\mathrm{OH}}^{-}$ ion conductivity of AEM, by using the Nernst-Einstein equation. However, the transportation of ${\mathrm{OH}}^{-}$ ions and chemical stability of AEM are complex. Moreover, chemical degradation reactions lead to the dynamic breakdown of working AEMs. Classical all-atom MD and coarse-grained MD simulations are not appropriate for the simulation of chemical degradation reactions and transportation of ${\mathrm{OH}}^{-}$ ions, as they cannot capture the forming and breaking of chemical bonds. The rapid development of the QM/MM technique results, in the foundation of ab initio MD and reactive MD, can simulate chemical degradation reactions and transportation of ${\mathrm{OH}}^{-}$ ions. Ab initio MD seems to be an optimal method, for exploring such reactions. There is, also, a quest to simulate large systems. Researchers are trying to build and simulate large AEM systems, including the QA head groups and backbones, via the coarse-grained MD. In addition, researchers, also, have developed models based on continuum physics for entire AEMFCs, using engineering correlations and ignoring the chemical structures of AEMs.

It is clear that in molecular & materials modeling, there is not a single modeling method that is omnipotent to solve all the problems. However, it might be possible to integrate different methods into a so-called multi-scale modeling approach, to study the chemical stability and transportation of ${\mathrm{OH}}^{-}$ ions of AEM at the microscopic level, phase separation at the mesoscale level, and device performance at the system level.

As remarked by Yang and Tarascon [214], in a commentary article, system-level planning of theoretical and experimental efforts is, increasingly, important for the development of modern materials science, and researchers have to pay considerable amounts of attention to studying the interface between individual components within a device or system. The modeling and simulation study of electrolyte and electrode interface in AEMFCs are important for the future, to study the chemical stability and transportation of ${\mathrm{OH}}^{-}$ ions in detail. In addition, most of the DFT, ab initio MD, classical all-atom MD, and coarse-grained MD studies reviewed in this work correspond to AEM-related problems at equilibrium. However, the external voltage is applied during the working mode of AEM, in reality. Modeling and simulations towards non-equilibrium situations are needed, to mimic real AEM environment.

In the future, the rapid development of physics-based and data-driven models [64,215,216], physics-informed machine learning methods [217], and others could advance the study of advanced energy materials, including lithium-ion batteries and fuel cells. In closing, we hope that this review has fulfilled the goal of introducing current molecular modeling methods and their recent applications to the AEM-based fuel cells research community.

## Figures and Tables

**Table 1:** Recent DFT-based computational studies on AEMs.

QA Head Group	Functional Used	Basis Set	Solvation Model	References
Imidazolium	B3LYP	6-311++G (2d, p)	PCM	[25,108]
Guanidimidazolium	B3LYP	6-311++G (d, p)	PCM	[109]
Imidazolium	B3LYP	6-311++G (2d, p)	PCM	[110,111]
Phenyltrimethylammonium	B3LYP	6-311++g (2d, p)	PCM	[112]
Benzyltrimethylammonium	B3LYP	6-311++G (2d, p)	PCM	[113]
Tetraalkylammonium	B3LYP	6-311++G (2d, p)	PCM	[114,115]
Imidazolium	B3LYP	6-311++G (d, p)	PCM	[116]
Various QA head groups	B3LYP	6-311++G (2d, p)	PCM	[94]
Vinyl benzyl	GGA-BLYP	—	COSMO	[117]
Trimethylammonium	B3LYP	6-311++G (d,p)	PCM	[50]

**Table 2:** Recent ab initio MD studies on AEMs.

AEM Structure	Function Used	References
Trimethylammonium-based polystyrene	GGA-BLYP	[124]
Trimethylammonium-based graphene bilayer	BLYP	[40,41,42,43]

**Table 3:** Recent all-atom molecular dynamics studies on AEM-related systems.

AEM Structure	Force Field Used	References
Various quaternary ammonium head groups	OPLS	[166]
Imidazolium grafted PPO chain	CHARMM	[167]
Functionalized Poly(phenylene oxide) AEM	ReaxFF	[168]
Four model AEMs with different functional groups	ReaxFF and APPLE&P	[169]
Imidazolium head group based poly(2,6-dimethyl-1,4-phenylene oxide)	COMPASS II	[140]
Four types of Polyvinyl butyral (PVB)-based polymer chains	AMBER	[170]
poly(vinyl benzyl trimethylammonium) (PVBTMA) in water with hydroxide ions	OPLS-AA and SWM4-NDP	[39]
Ethyl imidazolium functionalized poly(arylene ether sulfone)	COMPASS II	[171]
Fluorenyl group based poly(arylene ether sulfone ketone)	CVFF	[172]
Poly(ether ether ketone) (PEEK) polymer in water with hydroxide ions	COMPASS	[173]
Hydroxide transport in polysulfone-tetramethylammonium (PSU-TMA) membranes	DREIDING+rigid TIP5P	[174]
Quaternary-ammonized polysulfone vs. sulfonated polysulfone	DREIDING	[51]
Tetraalkylammonium-functionalized norbornene derivatives	(not mentioned)	[90,175]

**Table 4:** Recent CGMD studies on AEM-related systems.

System-of-Interest	Briefly on Simulation Methods	Reference
Properties of polyphenylene oxide tetramethylammonium (PPO-TMA)-based AEM	DPD	[177]
PPO/TMA-based AEMs: Effect of polymer architecture	united atom, LAMMPS	[195]
PPO/TMA-based AEMs: Exploring side-chain designs	DPD, DL_MESO	[196]
PPO/TMA-based AEMs: Hydrophobic and ionic interactions	CG model development, LAMMPS	[197]
Electrochemical performance of poly(ether ether ketone) (PEEK)-based AEM	CG model, based on the Martini FF	[198]
Polystyrene-b-poly(ethylene-co-butylene)-b-polystyrene (SEBS)-based AEMs	DPD, LAMMPS	[52,199]
Morphology of SEBS-based ionomers	DPD, LAMMPS	[200]

## Data Availability

Not applicable. This is a review article. The data that supports the original findings of the cited research studies are available within the cited articles.

## Abbreviations

The following abbreviations are used in this manuscript:

AEM	Anion Exchange Membrane
AEMFCs	Anion Exchange Membrane Fuel Cells
AFCs	Alkaline Fuel Cells
CGMD	Coarse-Grained Molecular Dynamic
CL	Catalyst Layers
DFT	Density Functional Theory
DPD	Dissipative Particle Dynamics
GDL	Gas Diffusion Layers
EFC	Enzymatic (Bio)Fuel Cells
DMFC	Direct Methanol Fuel Cells
HL	Hydration Level
HOR	Hydrogen Oxidation Reaction
IEC	Ion Exchange Capacity
FC	Fuel Cell
MCFCs	Molten Carbonate Fuel Cells
MD	Molecular Dynamics
MEA	Membrane Electrode Assembly
ML	Machine Learning
MM	Molecular Mechanics
${\mathrm{OH}}^{-}$	Hydroxide ion
ORR	Oxygen Reduction Reaction
PAFCs	Phosphoric Acid Fuel Cells
PEM	Proton Exchange Membrane
PEMFCs	Proton Exchange Membrane Fuel Cells
PEEK	Poly(Ether Ether Ketone)
PPO	Poly(P-Phenylene Oxide)
PS	Polysulfone
PVB	Poly(Vinyl Benzyl)
QA	Quaternary Ammonium
QM	Quantum Mechanics
$S_{N}2$	Nucleophilic Substitution
SEBS	Polystyrene-B-Poly(Ethylene-Co-Butylene)-B-Polystyrene
SOFCs	Solid Oxide Fuel Cells
